# Benefits and challenges with gamified multi-media physiotherapy case studies: a mixed method study

**DOI:** 10.1186/s40945-019-0059-2

**Published:** 2019-05-17

**Authors:** Doris Yin Kei Chong

**Affiliations:** 1 Department of Educational Research, County South, Lancaster University, Lancaster, LA1 4YD UK; 2 The Hong Kong Polytechnic University, Kowloon, Hong Kong

**Keywords:** Gamification, Multi-media, Physiotherapy education

## Abstract

**Background:**

The use of gamification in higher education context has become popular in recent years with one aim of enhancing learning motivation, yet, it is unknown how physiotherapy students perceive gamified education experience. Using gamification together with multi-media patient case studies, this study explored whether and how gamified education motivated physiotherapy students’ learning. It also investigated how other factors such as class design and mechanics affected gamified experience.

**Method:**

Six case studies in the subject Neurological Physiotherapy were transformed from paper-based cases to multi-media cases built by iSpring suite 8.1. Simulated, real or animated clients were used. Gamification mechanics such as leaderboards, scoring and prioritisation were embedded in the case studies. These gamified case studies were used in classes with Year-3 students enrolled in this subject. After taking these classes, 10 students participated in two focus groups and 32 students responded to a survey to share their experiences and perceptions on this pedagogy.

**Results:**

Results showed that students perceived gamified education as motivating since this satisfied their competence and social needs and enhanced their self-efficacy. In addition, authentic patient videos, class activities that allowed conflict resolution and reflection, and the use of leaderboards were enablers in this gamified experience.

**Conclusion:**

Future gamified education in physiotherapy can provide authentic experience through class designs and gamification mechanics to foster learning motivation. A suggested mapping of gamified lessons for physiotherapy education is provided based on the results of this study.

## Background

Learning is an inherently human activity that involves many complex active and interactive processes. Motivation appears to be a key driver to both initial and ongoing learning, as well as improved learning outcomes [1–4]. Gamification, or the use of game elements in non-game contexts [5], promotes achievement, challenge, goal, competition and collaboration to learning [6], which in turn motivates learners [7]. Gamification is thought to enhance motivation and engagement through three levels of processes: cognitive, psychological/emotional and social [8, 9]. At the cognitive level, learners experience processes such as problem-solving and decision-making [10]. At the psychological/emotional level, learners’ positive emotions (e.g. feeling competent) with certain experiences would wire into their memories to enhance further learning of similar experiences [11, 12]. At the social level, interactions with other learners facilitate knowledge constructions [13]. Gamified education should be structured to promote these processes.

To promote the aforementioned processes, better conceptualisations of gamification are needed. Gamification mechanics are often classified by reward or process-tracking types; namely leaderboards, badges, points (or scores), feedback and prizes [7, 9]. Some educational gamification systems use one type of mechanics while others use a mix-and-match approach. Pedersen and Poulsen [9] found that feedback and points showed an increase in positive outcomes in terms of learning motivations, while other mechanics warrant further investigations. In addition, it is important to differentiate between game-based learning, gamification and serious game. Game-based learning is the use of games (digital or non-digital) as learning tools [14], while gamification does not necessarily include a game but embed game elements (such as competitions) in learning tasks [5]. The term serious game is sometimes used interchangeably with game-based learning as it applies to any game with a purpose other than pleasure; here learning fits into this rationale [8, 15, 16]. The focus of this study is on gamification rather than game-based learning and serious game.

Gamification has been applied across different disciplines in higher education, such as computer science, mathematics, language and health education [17–19]. Currently, there is a lack of literature describing or studying gamification in physiotherapy education. In a recent systematic review on gamification in health care education by Wang, DeMaria [20], only two out of 48 reviewed studies included physiotherapy students as participants. This paucity warrants investigation in the use of gamification in physiotherapy education given its reported benefits on learning.

In the institution where this study was held, a significant portion of physiotherapy class time is dedicated to case-based learning. Case-based learning is a pedagogy where students integrate and implement knowledge learnt from basic science courses to an authentic patient care scenario [21]. The use of paper- or text-based cases followed by class discussion is a common practice in this study context. While providing real life patient information, teachers’ experiences attested that this common method may not provide students with the full picture of a patient’s problem because it is difficult for students to visualise and conceptualise clinical problems. Moreover, paper-based information appears dull and is difficult to motivate students. Using multimedia virtual patient cases combined with gamification can potentially overcome these disadvantages by embedding fun and interest in learning.

In relation to the above identified research gap and current pedagogical drawbacks, this paper contributes to the knowledge of gamification in physiotherapy education in several ways. First, a description of designing and implementing virtual patient case studies with gamification is provided. Second, physiotherapy students’ perceptions on gamified education experience as a motivational tool is explored. The implication is to provide a suggested mapping of gamified classes/experience used in physiotherapy education to promote motivation in learning.

### Gamification in higher education

Gamification has been gaining popularity in different fields including education [9, 17]. Various higher education disciplines reported using gamification in their curriculum, courses or a portion of the courses. Among all, computer science and information technology disciplines frequently use gamified education [17]. This is not surprising as gamification is mostly related to or perhaps easier to apply through computer games. Nonetheless, other disciplines using gamification include mathematics, communication/multimedia, medicine/biology/psychology, languages and other miscellaneous course [17–19].

The use of gamification in higher education is commonly found to improve motivation/self-efficacy [12] and students’ knowledge or performance [19, 22]. In fact, learner motivation is likely to be the primary reason for employment of gamification, as motivation is thought to be directly related to engagement and performance [23]. Claims of increased motivation and engagement through gamification have been criticised for their poor measurement [17]. The criticism is supported by Sailer, Hense [24] and this prompted them to conduct a study to map gamification elements/mechanics with various types of psychological needs behind motivation in a gamified environment. Although the participants of this study were not students in higher education, results shed lights on the inadequacy of mechanisms behind how gamified education motivated learners, as well as the importance of matching game mechanisms to the context.

With gamification being a new and novel concept in higher education, there is a need to understand the design, implementation, effect and mechanism of gamified education. Dichev and Dicheva [17] conducted a comprehensive review on the current status of gamification literature pertaining to higher education context. They found that literature on this topic were very diverse with regards to disciplines, gamification designs, goals, outcome measures and theories involved. This conclusion was somewhat concurred by Pedersen and Poulsen [9] where some gamification mechanics were found to have more positive effects than others; yet multiple questions related to how to frame gamification activities for optimal outcomes remained unanswered. Optimal design appears to be context-based and there may be a difference in terms of motivation between individual and group activities. In addition, studies on how gamification motivate learners are lacking [25]. With the diversity on possible theoretical frameworks underpinning gamification, some researchers even proposed gamification as a separate learning theory [26].

To conclude, although published studies on gamification within higher education appear adequate, and gamification appears to have the potential to positively affect learning in certain disciplines, better conceptualisations mapping between designs and mechanisms require further exploration.

### Gamification in health care education

Health care education is one discipline where gamification is applied within higher education; although, published literature is not as abundant as compared to other disciplines. In particular, literature on the use of gamification in holistic patient management (especially physiotherapy) is sparse. A rare example of this is by Johnsen, Fossum [27] that investigated the use of gamification to teach nursing students holistic clinical reasoning and decision-making skills. Study participants perceived the experience as clinically relevant and found this induced a level of realism to their learning. Gamification has also been found to improve engagement by embedding leaderboards and tournament prizes in medical simulated education [28], as well as improve knowledge and learners’ satisfaction in occupational therapy students [29].

Synthesizing results from limited literature in health care education, the current use of gamification in this discipline is quite heterogeneous. Some focus on a particular skill or knowledge content while some focus on a holistic case management. While others embed gamification concepts in a computer game; however, gamification mechanics were insufficiently described. Furthermore, some published works only described the design and implementation of games and gamification [16, 30] without any evaluative findings. Looking at this information, this current study attempts to address some of the gaps by including a larger sample size (100 students in gamified classes) and a holistic case management with gamification embedded. In addition, it provides a detailed description on gamification design and implementation, a preliminary evaluation on students’ perceptions of gamified experience, as well as a suggested mapping of design for future use.

### Motivation in gamified learning

As Sailer, Hense [24] stated, the main goal of using gamification in education is to motivate learners. Specifically, the essence of using games or gamification lies in promoting intrinsic motivation [31]. Intrinsic motivation in learning is defined as learning for pleasure and satisfaction as compared to for grades (extrinsic) [32]. It is intrinsic motivation that educators and researchers are interested in promoting because it drives behavioural change [33].

Gamification appears to promote intrinsic motivation through satisfying human’s psychological/emotional needs. Gamification helps to satisfy “competence need” by using badges, leaderboard and performance graphs in gamification [24]. Zarzycka-Piskorz [31] also found it is the winning or getting a reward that motivates learners. Furthermore, “social need” is met by using meaningful tasks/stories and teammates in gamification [24]. This point is concurred by Cheong, Filippou [11] that social element and feedback in gamification are possible motivating factors for students.

Another emotional need is self-efficacy, as it positively relates to motivation of learning and academic performance [34]. Self-efficacy is the belief of one’s ability to succeed in a task [35]. If a learner believes in his/her own ability to understand or master a task, he/she is likely to be more motivated in learning [34]. In gamification in higher education, this phenomenon is proven to be true by both Banfield and Wilkerson [12] and Harrold [10] where improved self-efficacy can change students’ learning habits. In addition, Feng, Jonathan Ye [36] reported that self-efficacy (together with self-presentation and playfulness) can facilitate participations in gamification.

Richter, Raban [37] found that gamification enhance self-efficacy through performance attainment, verbal persuasion, observation of other players and social influence. Successful performance, immediate feedback on performance, seeing similar performance of others and earning (social) reputation or recognition are all factors in gamification that could enhance self-efficacy [37].

As such, when designing and implementing gamified education, it is then important to consider the tasks or activities that can promote intrinsic motivation through addressing the various needs as described above.

### Aims of this study

The main goal of this mixed method study was to explore how physiotherapy students perceived gamified learning experience, especially in the area of motivation. Gamification is considered a multimedia pedagogy; however, the aim of this study was to map students’ experience for further study and therefore exploring how multimedia gamification helped with learning was not the goal. As such, the research questions for this study were:

1. What are physiotherapy students’ perceptions on their gamified education experience?
  - Was it motivating?
  - In what way did this experience motivate their learning?
2. What are the specific elements (e.g. gamification mechanics, case study videos, class activities) in gamified classes that influence their learning experience?
  - In what way did these elements motivate their learning?

## Methodology & Methods

### Study context and procedures

The project of including gamified virtual case studies was conducted within the course Neurological Physiotherapy II during the second semester of an academic year at a university in Hong Kong. This is a compulsory course in the Year-3 Bachelor of Science (Hons) in Physiotherapy curriculum. As described in the Background, the course includes lectures, tutorials and practical sessions as delivery modes and various pedagogies have been employed, such as didactic, hands-on and case-based learning. It is worth noting that not all course components were gamified; only six tutorials taught by the author were used for this project. In addition to adding gamification, three out of the six tutorial classes were switched from using a paper-based case study format to including virtual patient videos. The three virtual patient cases employed different “types” of patients – simulated, animated and real. The simulated patient case used actors to play the patient role, while the animated case used graphical animation in the case video. The real patient case, as the name implied, was filmed with a patient with real neurological condition. In addition, two out of the three paper-based classes were not using a patient case format and class activities involved searching and presenting factual disease information.

### The gamification building team

**Fig. 1:**
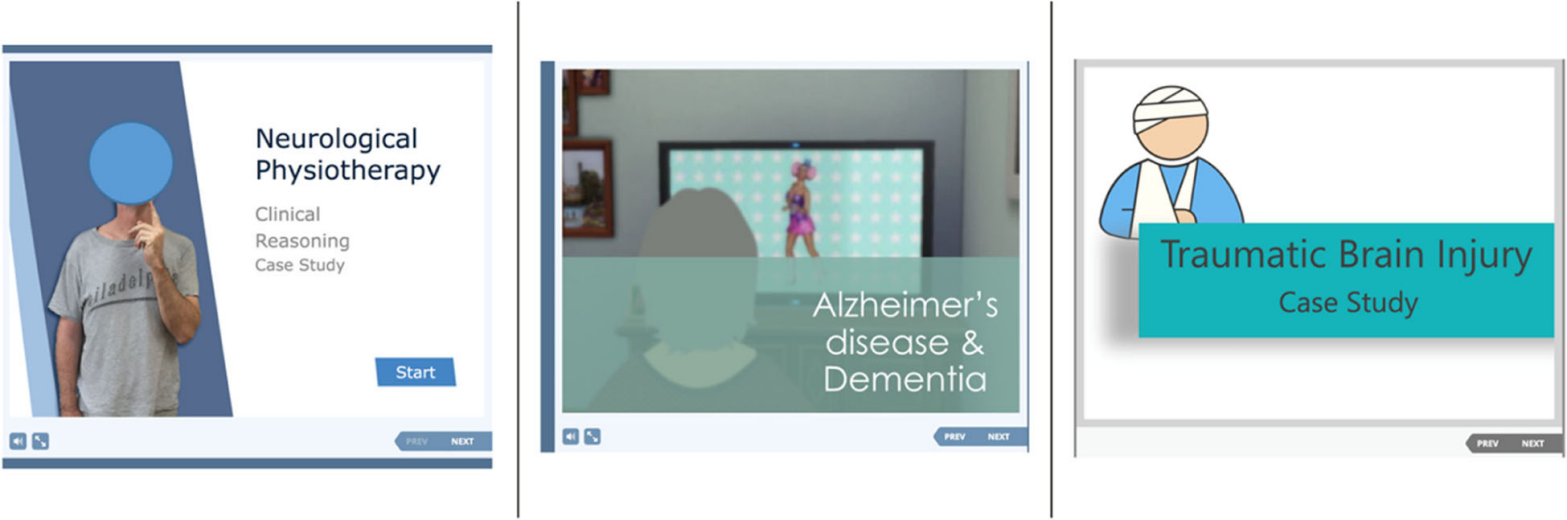
Title pages of the three gamified virtual patient cases

The project team consisted of an expert on game-based learning from School of Design, an expert on instructional design from Educational Development Center and two Physiotherapy content experts (including the author) from the course teaching team within the university. A project assistant who was a student in the Multimedia Design bachelor programme was hired to provide design ideas and to build the cases/gamification mechanics. This project was funded by the Advisory Committee on eLearning Learning and Teaching Development Grant 2015–2017 at the university. Planning, design and building of the three gamified virtual patient case studies with input from all parties occurred between July and December prior to the implementation semester. The cases were built mainly using iSpring Suite 8.1 and housed on the Blackboard course site. A prototype was developed and feedback were collected from ten Year-4 students who had taken the course in the previous year. Further case modifications were done based on the feedback from the project team and upper year students. Figure 1 shows the title pages of the three iSpring cases.

### Gamification processes and learning aims

The six gamified tutorial sessions happened between February and March of an academic year. The cohort (*n* = 100) were divided into four tutorial groups where the same set of learning activities (e.g. case discussions, quizzes, treatment demonstrations) were repeated. Each tutorial session was 2-h long. During the first gamified session, the author asked each tutorial group to form four teams and to remain in those teams throughout the gamified classes.

**Fig. 2:**
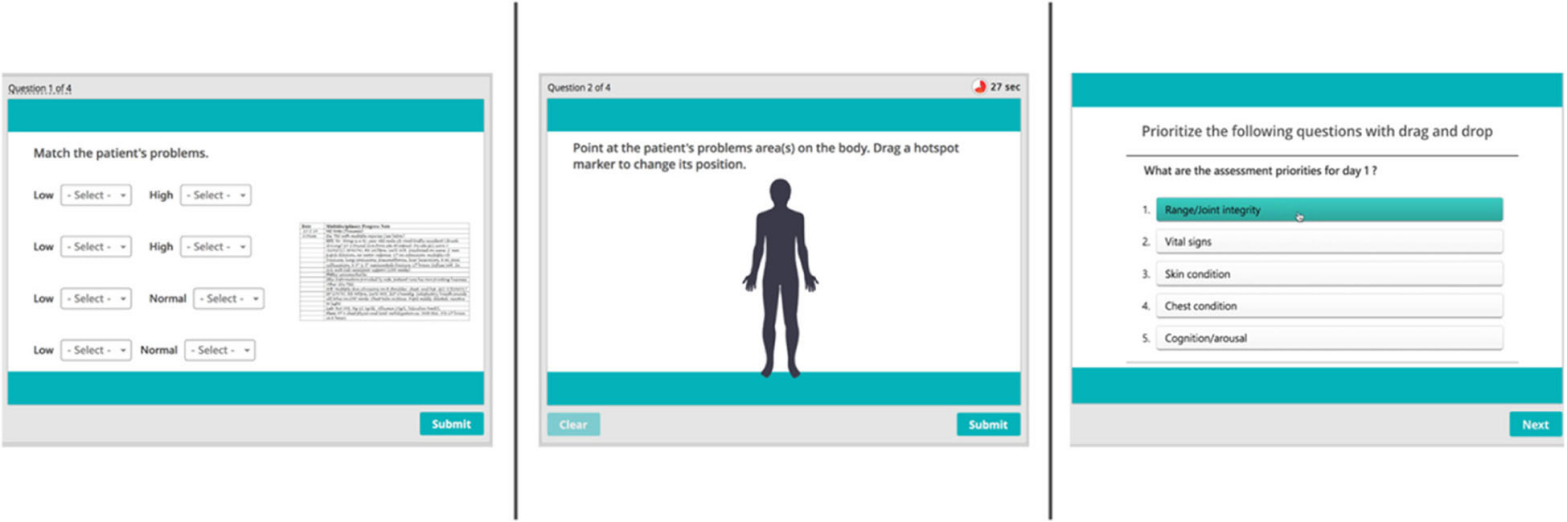
Examples of gamified activities embedded in the “Traumatic Brain Injury” case study. Activities from left to right: Matching, hotspot, drag and drop for prioritisation

Although each session has its own set of learning objectives depending on the disease covered in that session, the overall learning objectives of case studies focus on students’ ability to explain or justify physiotherapy assessment and treatment choices, an authentic case management experience for students. A combination of activities was embedded in the six classes in order to reach these objectives, e.g. quizzes using built-in iSpring functions and other online websites such as Kahoot, prioritisations using the drag and drop function, hotspots, discussions, presentations and demonstrations. Figure 2 shows examples of activities embedded in the case study of “Traumatic Brain Injury”.

**Fig. 3:**
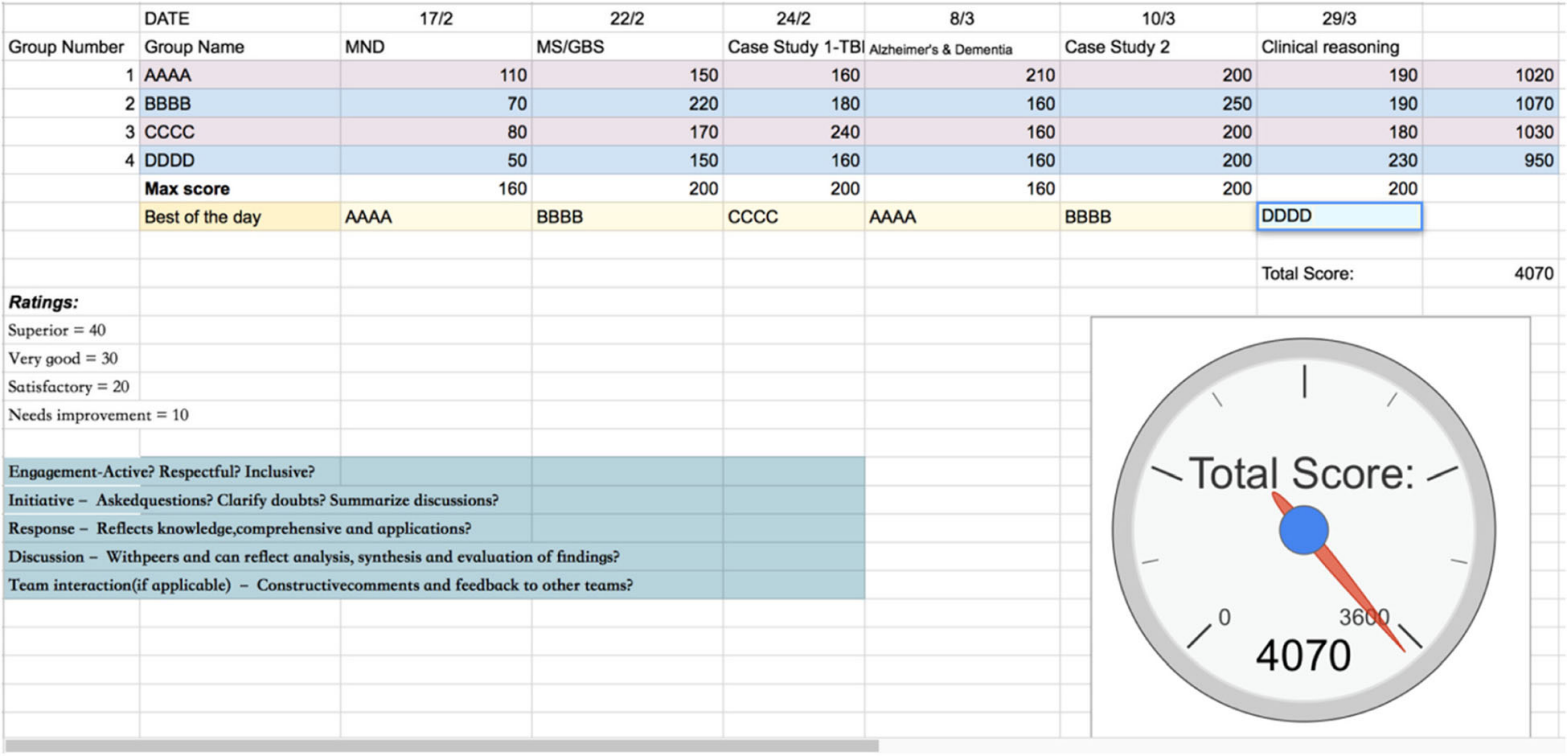
Score/Leaderboard (with team name modified to AAAA, BBBB, CCCC and DDDD). Each team may get a score between 40 and 200 per session depending on the rating scale on the left hand-side of the figure. The last column shows the final score of that team after six sessions. The score clock shows the accumulated total scores of all teams

At the end of each gamified class, the instructor gave a score for each team based on their interaction and participation. The team that demonstrated the most active and fruitful discussion and peer interaction would get the highest score. Each tutorial group also voted for the “Best Team of The Day” according to team performance and this team earned an extra 50 points. A scoreboard/leaderboard (see Fig. 3) was posted online so students could keep track of their progress. At the end of the six gamified classes, the team with the highest score in each tutorial group was awarded a “goodie bag” (consisted of stationaries). As a way to motivate students to participate, students were told they needed to achieve an accumulated 3600 points in order to unlock a case study video to help them with examination preparation. The case study was in fact available to all students after the end of these gamified classes. In brief, this project employed leaderboards, prizes, achievements and progress tracking as gamification mechanics with a goal to spark competition and motivation [7].

### Participants and methodological tools

The study’s participants included all Year-3 physiotherapy students (*n* = 100) who were enrolled in the course. After the completion of all gamified classes, all students were invited to voluntarily and anonymously complete an online post-experience survey. In addition, twelve students were purposefully invited by email to join one of the two focus groups conducted one week after the last gamified class was over. These twelve students were recruited from all four tutorial groups and from different teams in order to get a better and more equivalent representation of experiences. Also, they were observed to be willing to share their thoughts and ideas in class and thus believed to have a unique contribution in this study [38]. The participants’ information and consent form were attached to the invitation email and students were free to join or opt out of the interview. Although the researcher in this study was also the teacher of the gamified classes, students were reassured that their participation in the focus group would not affect their academic results. In total, seven male and three female students volunteered to join.

Focus groups allow participants with a similar background to interact and share opinions on the topic of interest [39]. Through this interaction, rich information and meaning of experience and perception are available [40]. Since the aim of this study was to explore students’ in-depth perceptions and meanings of this gamified education experience, focus group is deemed appropriate. On the other hand, surveys allow more students to provide their views on this gamified education experience [41]. Survey data is more concrete, easy to analyse and can supplement the results gathered from focus groups [41]. This triangulation of data allows for a broader representation and understanding of students’ experience.

### Data Collection & Analysis

The two focus groups were conducted in Cantonese by the author, each lasted for about one hour and was audio-recorded. An interview guide with questions (see Appendix 1) was developed to help the facilitator and participants to stay focused during the discussion, but allow for follow-up questions as needed. All audio recordings were translated and transcribed into English first by the project assistant and cross-checked by the author. The author undertook thematic analysis based on the five steps suggested by Giorgi [40]: (1) collection of verbal data (by focus groups) (2), reading of data (English) (3), dividing of data into parts (meaning units) (4), organisation and expression of raw data into disciplinary language (coding that fits into our discipline) and (5) summary of data to communicate with scholarly community (themes).

The post-experience survey consisted of 12 items and was used to gather general perception and satisfaction on the virtual patient cases (see Table 1 for survey items). The question items were developed based on the research questions of this study and the author’s knowledge of satisfaction survey. Participants rated their level of agreement on a 5-point Likert Scale in each item, where 1 = strongly disagree and 5 = strongly agree. The combined percentage of “agree” and “strongly agree” is reported and is considered as positive results.

**Table 1:** Post-experience survey items and results

Survey Items	% of agreed and strongly agreed
1. The instructions for the online virtual patient cases are clear.	93.75%
2. The case content is helpful to achieve the learning objectives.	93.75%
3. The questions/activities are useful for assessing my level of understanding of the case content.	93.75%
4. The difficulty level of the questions/activities is appropriate.	90.625%
5. The length of the cases is appropriate.	93.75%
6. I have good understandings of the content after the cases.	84.5%
7. The online virtual patient cases are useful for enhancing my motivation.	90.625%
8. The questions/activities in the cases are engaging and interactive.	93.75%
9. The use of gamification (e.g. narration storytelling, decision making for virtual patient) is useful.	93.75%
10. The use of multimedia (e.g. teaching videos, animation, pictures) in the online cases is effective.	90.625%
11. The feedback from the questions/activities are clear.	84.375%
12. Overall, I enjoyed the online virtual patient cases.	96.875%

## Results and Discussion

After several invitations and reminder emails, 32% (*n* = 32) of the class completed the post-experience survey. Overall, 96.875% of respondents agreed or strongly agreed that they enjoyed the virtual patient case study experience and 93.75% of them agreed or strongly agreed that the use of gamification in class is useful. Table 1 shows the details of survey result.

The focus group discussions revealed two major themes related to the research questions: (1) Gamification experience enhances learners’ motivation and (2) Gamification experience depends on game mechanics and case study designs. They are discussed below together with relevant results from the survey.

### Gamification experience enhances learners’ motivation

Over 90% of survey respondents agreed or strongly agreed that the gamified classes were motivating. Nearly 94% of respondents thought the activities were interactive and engaging. Qualitative data revealed that students generally described the gamified classes as entertaining, playful, pleasant, authentic and as an encounter that can potentially change their thinking process. These general feelings are overall in line with previous research findings from other health education disciplines where participants perceived “fun and pleasant” [42], “realistic” [27] and “practice-changing” [42] experiences with their gamified education.

Sense of happiness and success is attributed as a potential motivator behind gamified classes:

“I think the classes were playful; everyone was involved” (Gp1S3)

“I think we were happy…” (Gp1S4)

“I felt a sense of success” (Gp1S3) and concurred by Gp1S4 that “yes, it was a very high sense of success”

Sense of happiness and success can be viewed as a form of “social need” and “competence need” respectively, as described by Sailer and colleagues [24]. It is reasonable to believe that these two emotional needs underpinned motivation behind our gamified education. In addition, “being recognised” in gamified classes was mentioned by our students and it can be a form of self-efficacy described by Banfield and Wilkerson [12]. Specifically to our context, this form of self-efficacy came about because of a lack of acknowledgement in usual classes:

“How many times do we (students) get recognition in class? Very few…..The score is a form a praise….at least we praise ourselves.” (Gp1S2).

Similar to Banfield and Wilkerson [12], our gamified case study classes offered students a continuous learning process where students had to form and reform their ideas based on authentic patient scenarios and active discussions among themselves. This process was grounded by students’ experience (playful, success and recognition) of enhanced motivation and active engagement. All in all, this learning process allowed our students to experience the potential of thought process and knowledge change through enhanced motivation:

“I can be better prepared and know my deficits in clinical reasoning…the entire process is different…I can think of the entire situation and the process…” (Gp2SB)

### Gamification experience depends on game and design mechanics

Over 90% of students thought the use of multimedia gamification classes was effective; however, this experience depended upon class and game designs, such as video designs, class activities and gamification mechanics.

#### Video designs

When comparing to text-based case studies, students generally praised virtual video case studies (using real person) as more vivid, remarkable, and useful to enhance their observational skills, as illustrated in the extracts below:

“…with only paper-based cases, we don’t understand the problem…we cannot visualize the presentation.” (Gp2SE)

“…it is important for clinical reasoning…if I can’t observe I don’t know what to treat…” (Gp1S2)

With regards to the types of virtual patient, students unanimously preferred videos with either real patient actors or real patients, as compared to animated patients because of its questionable authenticity. This finding is in contrast to other studies that have reported beneficial learning effects using virtual 3D animations (e.g. Second Life) [43, 44]. The difference may be due to the 3D and interactive nature of Second Life, which was not present in our animated patients.

#### Class activities

Learning activities embedded in gamified classes influence how students perceive their gamification experiences. Peer teaching in the form of presenting factual information appeared to be less-received by students, but students appreciated observing demonstration of treatment skills and ideas:

“During presentation, I don’t know if they are presenting the right or wrong information…this is the most important thing.” (Gp1S4)

“…it is good that some students think from a different angle…I’m surprised to see their demonstrated methods, and realized their ways are better than mine.” (Gp2SB)

Previous studies suggested that the effect of peer teaching is questionable and one reason being the accuracy of peer assessment and feedback [45]. Although small classes are generally preferred in peer teaching [46], the type should be carefully selected. Listening to presentation is perceived as a boring, one-way activity while observing and commenting on treatment skills/ideas gives students opportunities to interact and transact between people and environment. In addition to the preferred format of peer teaching, the instructor’s role is influential. In this study, the role centred around instruction, guidance and feedback:

“Before watching the videos, perhaps state what we need to do, e.g. assessment or treatment…” (Gp2SA)

“If you give us a bit more guidance…you can explain a bit more how we can think from different angles and perspectives…” (Gp2SB)

“Many questions (in the cases) were not explained and there was no follow-up” (Gp2SD)

With regards to feedback, explicit guidance and expectation from the instructor are perceived as effective in the eyes of students [47]. This is similar to what our students had suggested and revealed from the post-experience survey, in which only 84.375% agreed or strongly agreed that feedback embedded in the case studies was clear. Feedback on performance and progress would have enhanced self-efficacy in our students as feedback serves to position students towards their goals in gamified education [37]. Thus, peers’ and instructors’ interactions should be precisely mapped in order to facilitate or motivate students.

There is a paucity of literature on best practice in gamified classroom design. Baldeon, Rodriguez [48] suggested a learner-centred design framework for gamified education, where the learners and the context need to be understood, and activities should follow a fun-activity-loop. Activities should be based on the specific learning style(s) of learners. The actualisation of this framework in class warrants further development and research.

#### Gamification mechanics

Leaderboards, scores, prizes and teammates were mechanics used in our gamified classes. Students found leaderboards the most motivating mechanic; though, the effects of leaderboards could be further enhanced by increasing its visibility:

“I realised our tutorial group has the highest score among all…I think we are smart…I will work even harder to stay being the best.” (Gp1S2)

“If it is more visible, for example, on blackboard rather than a separate link, it will be better…it can create more noise.” (Gp1S3)

Leaderboards give a sense of achievement (or lack of achievement) which in turn motivates participants to work harder in order to reach a certain level of accomplishment. Pedersen and Poulsen [9] and Sailer, Hense [24] concluded similarly in their studies that leaderboards improve motivation and academic performance. This is likely related to what Sailer, Hense [24] described as “competence need” underlying enhanced motivation in gamified education. In addition, leaderboards give students a visualisation of their performances. Students can observe their own performance and also others. This visualisation provides students with a form of judgement and in turn promotes self-efficacy [37].

Despite the positive effect of leaderboards demonstrated in this research, their use is not without critique. Mekler, Bruhlmann [49] stated that although leaderboards, points and levels improve performance, they seem to have no effects on intrinsic motivation. Hanus and Fox [50] even reported harmful effects on intrinsic motivation of various gamification elements in their longitudinal study. Nonetheless, the use of leaderboards seems to align with goal-setting theory where learners consistently set their learning goals at high end and this is perceived as a way to enhance intrinsic motivation [51].

Interestingly, our students expressed that scores earned in gamified classes did not motivate them to learn, unless scores were included in the final course grade. This is in contrast to the findings reviewed by Pedersen and Poulsen [9]. In fact, this is also controversial to how our students appreciated the use of Kahoot (a free game-based learning platform) in gamified classes, where they scored based on their speed and accuracy of responses to questions asked. Students expressed that Kahoot was a good tool for checking knowledge and stimulating participation and that was why they enjoyed it. Perhaps it is the process of playing Kahoot rather than the score (outcome) that motivated students, through recognising mastery of knowledge [37].

Prizes were sparsely mentioned in the focus group discussions; hence, it is difficult to understand how this mechanic is perceived. On the other hand, team dynamics were repeatedly mentioned, yet feelings towards working in teams were mixed. The reason behind, as expressed by this student, could be:

“most of my teammates are not this type of students (learn by themselves)…it’s a bit difficult…the person who participated in Kahoot is the only one (within a team) who will prepare for class…” (Gp2SE)

This point is well supported in the literature. Sailer, Hense [24] stated that teammates influence social-related experience in gamified education. Pedersen and Poulsen [9] also stated that positive group dynamic, namely engagement and interaction among themselves, is critical to the applicability and success within gamification. It is therefore not surprising to find mixed perceptions on how our students perceive their team experience, when not everyone is interactive and active.

### Study implications

**Fig. 4:**
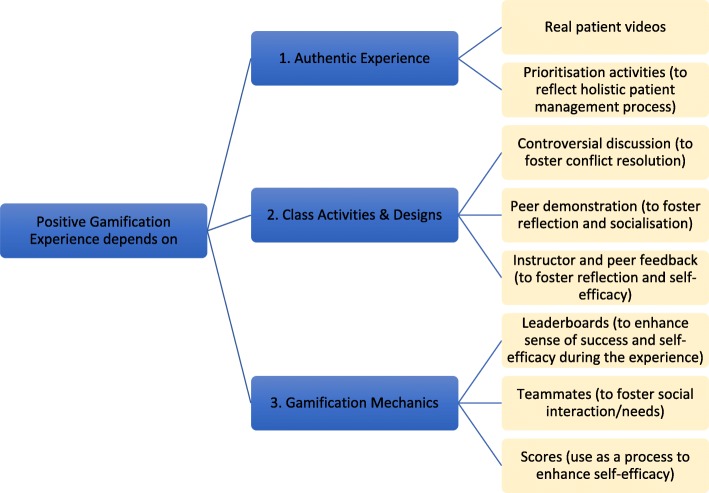
Suggested mapping of class designs and gamification mechanics that could enhance positive experience and motivation in gamified education. Positive experience depends upon authentic experience, class activities and designs and gamification mechanics. The yellow boxes indicate suggested activities and mechanics

Synthesising results from this study, several implications could be suggested for physiotherapy education. First, the opportunities of gamification depend upon authenticity of students’ experiences. Using real patient videos and learning activities (prioritisations, discussions and demonstrations) that mimic a real patient management process (forming and reforming ideas) in clinical settings can be one way to enhance authenticity. However, it doesn’t end here. Adding carefully considered class activities and gamification mechanics can be crucial to the success of promoting intrinsic motivation in an authentic learning process. Specifically, mechanics that induce senses of happiness, success and self-efficacy are perceived as important in our context. Leaderboards can enhance self-efficacy and sense of success, while feedback from instructors and reflections on own ideas (especially in controversial situations) can satisfy the social and cognitive needs of learning process. It implies that the design of gamified classes need to be carefully mapped to address the needs for promoting intrinsic motivation. Figure 4 shows a suggestion of this mapping that can be adopted in future physiotherapy education where the focus is on patient management process.

### Study limitations

The goal of this study was to explore physiotherapy students’ perception on gamified learning experience, in order to map future development of gamification in physiotherapy education. The study therefore did not investigate potential areas such as usability of gamified cases, learning effects and how this multimodality approach affects learning. It was not appropriate to measure learning effect because not all classes in the course were gamified. Any effect on overall learning performance cannot be attributed to gamification alone. In addition, not all gamification mechanics were included in this study. It is unknown if other mechanics might have contributed to learners’ perceived motivation. Furthermore, despite the assumption that gamification motivated learning, standardised objective measurements on motivation were not included. Low post-experience survey response rate might also have limited the interpretation of study results. Lastly, the suggested mapping may only apply to similar context (e.g. occupational therapy and nursing where patient management process is the key learning objective) but may not apply to other higher education disciplines if the goal is mainly for factual knowledge retention.

## Conclusion

This study explored how physiotherapy students perceived their learning experience with gamified classes. Gamification enhanced students’ motivation by giving them a sense of happiness, a sense of success and by fostering their self-efficacy. These are all factors that can promote intrinsic motivation for learning. The learning process was well accepted as it was authentic and was grounded in a motivating experience.

Gamification experience depended upon authenticity of virtual patient videos, class activities and mechanics used. Real patient videos were preferred over other types because of authenticity. Class activities that required conflict resolutions of ideas and transactions between people and environment helped with knowledge creation. Feedback on performance and progress was important to enhance self-efficacy.

In the research context, leaderboards motivated our students through visualisation of progress; though other mechanics warrant further investigation. Team dynamics influenced gamified education with active participation being the key to success to forming and reforming ideas/knowledge based on case information.

In summary, this study demonstrated favourable potentials of embedding gamification in physiotherapy education. Virtual patient videos should be authentic and activities should facilitate controversial discussions. Clear guidance and feedback from instructors are essential. Leaderboards can potentially motivate learners; however, team dynamics need to be fostered in order to achieve the optimal benefits of social interactions.

## Appendix 1

### Interview Guide

1. How were the classes with the use of online learning tools? What’s your overall impression?
  A. Immediate results and feedback?
  B. Facilitation during class?
  C. More concentrated in the case study?
  D. Learn from other groups?
2. Paper-based case study turned into gamified online cases. Which part(s) motivated you to learn?

A. Videos demonstration?
B. Videos scenario?
C. Animation?
D. Quizzes?
E. Decision making on treatment decision?
F. Points/Competition?

3. Are there any influences on your learning with the use of gamification?

A. Higher learning motivation?
B. Less stressful?
C. Clearer content?
D. Deeper impression?
E. More class interaction?

4. Which multimedia types do you think are most helpful? Why?
  A. Visualized the virtual case?
  B. More realistic?
  C. Reflecting on the real situation?
  D. More discussion with the media materials?
5. What are the barriers hindering learning and clinical reasoning using multimedia cases?
  A. Not enough feedback?
  B. Misunderstand the content?
  C. Insufficient discussion?
6. Any addition comments or suggestions?
